# Ligand Valency Affects Transcytosis, Recycling and Intracellular Trafficking Mediated by the Neonatal Fc Receptor

**DOI:** 10.1111/j.1600-0854.2006.00457.x

**Published:** 2006-06-29

**Authors:** Devin B Tesar, Noreen E Tiangco, Pamela J Bjorkman

**Affiliations:** 1 Division of Biology and California Institute of Technology Pasadena, CA 91125, USA; 2 Howard Hughes Medical Institute, California Institute of Technology Pasadena, CA 91125, USA

**Keywords:** apical, basolateral, FcRn, FcRn-GFP, heterodimeric Fc (hdFc), IgG, Madin–Darby canine kidney (MDCK) cells, transcytosis

## Abstract

The neonatal Fc receptor (FcRn) transports IgG across epithelial cell barriers to provide maternal antibodies to offspring and serves as a protection receptor by rescuing endocytosed IgG and albumin from lysosomal degradation. Here we describe the generation of polarized Madin–Darby canine kidney (MDCK) cells expressing rat FcRn (rFcRn) to investigate the potential requirement for ligand bivalency in FcRn-mediated transport. The rFcRn-MDCK cells bind, internalize and bidirectionally transcytose the bivalent ligands IgG and Fc across polarized cell monolayers. However, they cannot be used to study FcRn-mediated transport of the monovalent ligand albumin, as we observe no specific binding, internalization or transcytosis of rat albumin. To address whether ligand bivalency is required for transport, the ability of rFcRn to transcytose and recycle wild-type Fc homodimers (wtFc; two FcRn-binding sites) and a heterodimeric Fc (hdFc; one FcRn-binding site) was compared. We show that ligand bivalency is not required for transcytosis or recycling, but that wtFc is transported more efficiently than hdFc, particularly at lower concentrations. We also demonstrate that hdFc and wtFc have different intracellular fates, with more hdFc than wtFc being trafficked to lysosomes and degraded, suggesting a role for avidity effects in FcRn-mediated IgG transport.

The plasma membranes of epithelial cell barriers are segregated into two spatially and functionally distinct domains that serve as a means for complex organisms to distinguish between the external environment and the underlying tissue. A vital characteristic of such cellular barriers is their ability to selectively allow the passage of materials, such as ions, small molecules, peptides, lipids and proteins, either by passive or active (receptor mediated) transport mechanisms. The neonatal Fc receptor (FcRn), a class I major histocompatibility complex (MHC)-related protein that associates with the MHC light chain β_2_-microglobulin (β_2_m), mediates the transfer of maternal IgG across epithelial cell barriers to the fetus or newborn (1,2). In newborn suckling rodents, FcRn is expressed in the polarized epithelium of the intestine. The FcRn present at the apical surface of the intestinal cells binds to maternal IgG from ingested milk, transcytoses it across the epithelium and releases it into circulation from the basolateral cell surface (1). This process confers passive humoral immunity to the newborn during the first weeks of independent life. The difference in pH between the intestinal lumen (∼pH 6.0) and the bloodstream (∼pH 7.4) promotes the efficient unidirectional transport of IgG, as FcRn binds IgG at pH values ≤6.5, but not at neutral or higher pH (1,3).

The FcRn-mediated transport of IgG can also occur in the absence of a pH gradient. In gestating primates, IgG in the maternal bloodstream is transferred to the fetal bloodstream in a process that consists of passive uptake of IgG by syncitiotrophoblast cells, followed by transcytotic delivery to the fetal bloodstream on the opposite surface (4–6). In adult mammals, FcRn plays a key role in serum IgG homeostasis by protecting IgG taken up by vascular endothelial cells from a default degradative pathway (7–9). For both these functions, it is believed that IgG internalized at pH 7.4 via fluid-phase endocytosis is bound by FcRn in acidic endosomes. This results in FcRn–IgG complexes being transcytosed in the case of maternofetal IgG transfer, or returned to the cell surface rather than catabolized in the case of serum IgG homeostasis.

We previously described a structure-based hypothesis to account for the ability of cells to distinguish endosomes containing FcRn–IgG complexes destined for recycling or transcytosis from endosomes destined for a degradative pathway (10). The hypothesis suggests that an oligomeric ribbon of FcRn dimers bridged by the homodimeric Fc regions of IgG molecules, as seen in the crystal structure of an rFcRn/Fc complex (11), forms inside acidic trafficking vesicles. Formation of the oligomeric ribbon between the adjacent membranes of a tubular endosome could act as an intracellular trafficking signal, designating vesicles containing such complexes for entry into the transcytotic or recycling pathways. A requirement for the formation of the oligomeric ribbon is a homodimeric Fc or IgG molecule capable of bridging between FcRn proteins on opposing membrane faces. Consistent with this hypothesis, previous studies demonstrated that a heterodimeric Fc molecule (hdFc), which contains one FcRn-binding chain and one non-FcRn-binding chain, is less efficiently transcytosed across neonatal mouse intestine (12) and exhibits a shorter serum half-life (13) than a wild-type homodimeric Fc molecule (wtFc). These results indicate that two FcRn-binding sites on a ligand are required for the purposeful movement of vesicles containing FcRn–ligand complexes through the transcytotic and protection pathways. However, recent studies have demonstrated that human and rodent FcRn bind a monomeric ligand, serum albumin, with a similar pH dependency as the FcRn–IgG interaction, and that mice lacking either the FcRn heavy or light chain exhibit a shortened half-life for albumin in the circulation (14,15). These studies support a model in which FcRn acts as a protection receptor for a monomeric protein, albumin, as well as for dimeric IgG and Fc ligands.

Here we describe an *in vitro* system using transfected Madin–Darby canine kidney (MDCK) cells to compare the transport of dimeric and monomeric FcRn ligands. The MDCK cells expressing rat FcRn (rFcRn) transcytose Fc and IgG in both the apical to basolateral and basolateral to apical directions, consistent with previous studies of human FcRn (hFcRn) and rFcRn expressed in MDCK cells or other polarized cell lines (16–23). We do not observe specific binding, uptake or transcytosis of a naturally occurring monovalent FcRn ligand, rat albumin. We therefore used variant forms of rat Fc containing two, one or zero FcRn-binding site (24) to assess the effects of ligand valency on FcRn-mediated transport. We show that the presence of two binding sites on the internalized Fc is not strictly required for the transcytosis or recycling to occur, but a bivalent Fc with two FcRn-binding sites (wtFc) is trafficked more efficiently than its monovalent cognate (hdFc), particularly at lower concentrations. Analysis by confocal microscopy of wtFc and hdFc trafficking following internalization reveals that the two ligands have different intracellular fates such that more internalized hdFc than wtFc colocalizes with markers for early endosomes (EEA1) and lysosomes, consistent with quantitative studies demonstrating that more hdFc than wtFc is degraded after internalization. These results suggest that avidity effects play a key role in FcRn-mediated ligand transport.

## Results

### Functional expression of rFcRn in MDCK cells

Our laboratory previously described the generation of MDCK cell lines expressing rFcRn and an rFcRn-green fluorescent protein (GFP) chimeric protein (25). In the course of conducting new experiments with the previously described rFcRn-GFP-MDCK cell line, we discovered that we could not replicate some of the published properties of the cell line (26). It had been reported that the rFcRn-GFP-MDCK cells functioned in transport of Fc when ligand was added at both permissive (acidic) and nonpermissive (basic) pH values for the rFcRn–IgG interaction and that rFcRn-GFP fluorescence underwent a striking redistribution upon addition of ligand at either pH (25). In recent experiments, however, we found that the rFcRn-GFP-MDCK cell line, although positive by antibody staining for both the rFcRn heavy and light chains (data not shown), did not take up significant amounts of Fc or IgG at basic pH, and the distribution of rFcRn-GFP did not change upon ligand addition (26).

To resolve these discrepancies, we generated new MDCK cell lines stably expressing rat β_2_m (rβ_2_m) together with the full-length rFcRn heavy chain or with an rFcRn-GFP chimera in which GFP was added C-terminal to the rFcRn cytoplasmic tail. Drug-resistant transfected cells were screened by Western blot and by pH-dependent uptake of fluorescent rat Fc. A drug-resistant cell line transfected with an empty expression vector (vector-only MDCK) was used as a negative control. The rFcRn-MDCK and rFcRn-GFP-MDCK cells used in the present studies exhibited transepithelial electrical resistance (TEER) values of ∼250–300 Ωcm^2^ when grown as polarized monolayers on filter supports. The high TEER value is important, as the rFcRn-GFP-MDCK cell line described previously (25) was subsequently found to have low TEER values (∼50–75 Ωcm^2^), consistent with our later finding that the cells were leaky to radiolabeled ligands (our unpublished results).

**Figure 1:**
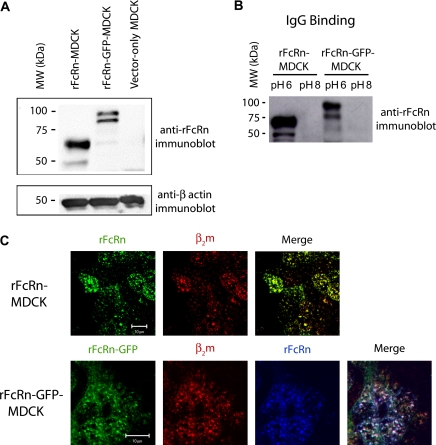
Expression of rFcRn and rFcRn-GFP in MDCK cells (A) Western blot using anti-rFcRn antiserum of detergent lysates from rFcRn-MDCK, rFcRn-GFP-MDCK and vector-only MDCK cells. Two bands, likely representing the mature and incompletely glycosylated rFcRn heavy chains, are detected in rFcRn-expressing cells, but not in control cells. (B) Western blot of detergent lysates after a pull-down with human IgG-Sepharose at pH 6 or pH 8. Bands corresponding to the rFcRn and rFcRn-GFP heavy chains are present in samples from pull-downs at pH 6. No bands are detected in samples pulled down at pH 6 and subsequently washed at pH 8 (data not shown). (C) Confocal images of rFcRn-MDCK and rFcRn-GFP cells stained with fluorescently labeled anti-rFcRn (green or blue) or anti-rβ_2_m (red) antibodies. Staining of rFcRn-MDCK cells shows that rFcRn-positive compartments colocalize with rβ_2_m-positive compartments, and staining of rFcRn-GFP-MDCK cells shows that fluorescence from GFP colocalized with fluorescence from both the anti-rFcRn and anti-rβ_2_m antibodies.

Expression of rFcRn and rFcRn-GFP in the newly generated MDCK cell lines was verified in cell lysates by Western blot. An anti-rFcRn antiserum detected two bands migrating with apparent molecular masses of ∼50 and ∼65 kDa in the rFcRn-MDCK cell lysate and ∼80 and ∼95 kDa in the rFcRn-GFP-MDCK cell lysate (Figure 1A). The upper and lower bands likely represent mature and incompletely glycosylated forms of rFcRn, respectively, as previously observed for hFcRn expressed in MDCK (16) cells and rFcRn expressed in rat inner medullary collecting duct (IMCD) cells (22). To verify IgG binding by rFcRn in MDCK cells, whole-cell lysates were incubated with human IgG-Sepharose beads at acidic or basic pH, and the bound proteins were eluted and analyzed by Western blot. The rFcRn and rFcRn-GFP were detected in IgG pull-downs conducted at acidic but not at basic pH (Figure 1B), although both proteins were detectable in blots of total cell lysates from rFcRn-MDCK and rFcRn-GFP-MDCK at both pH 6 and 8 (data not shown). Additionally, no rFcRn or rFcRn-GFP is detected in IgG pull-downs performed at pH 6 and subsequently washed at pH 8 (data not shown), demonstrating that both forms of rFcRn bind IgG at pH 6 and dissociate from the ligand at pH 8.

Confocal analyses of rFcRn-MDCK and rFcRn-GFP-MDCK cells showed intracellular compartments that are labeled by antibodies against the heavy and light chains of rFcRn (Figure 1C). The stainings for rFcRn and rβ_2_m are colocalized, consistent with proper association between the two chains, and both the anti-rFcRn and the anti-rβ_2_m fluorescence colocalize with GFP fluorescence in the rFcRn-GFP-MDCK cell line, indicating that GFP can be used as a marker for rFcRn expression in this cell line.

**Figure 2:**
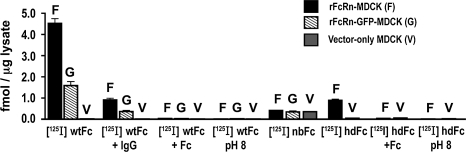
Endocytosis of rFcRn ligands by rFcRn and rFcRn-GFP expressed in MDCK cells Subconfluent rFcRn-MDCK, FcRn-GFP-MDCK or vector-only MDCK were incubated with radiolabeled Fc ligands (recombinant wtFc, hdFc and nbFc derived from rat IgG2a at a concentration of 20 nM) at pH 6 or pH 8 in the presence or absence of a 500-fold excess of unlabeled competitor protein, and levels of radioactivity were determined in cell lysates.

The ability of rFcRn-MDCK and rFcRn-GFP-MDCK cells to uptake wtFc, a recombinant rat Fc (24), was evaluated by a quantitative radioligand endocytosis assay. As shown in Figure 2, rFcRn-MDCK and rFcRn-GFP-MDCK cells internalized a significant amount of [^125^I]wtFc at pH 6 but not at pH 8. Internalization was saturable, as inclusion of the unlabeled wtFc or IgG reduced the uptake to background levels, and was also specific, as significant uptake of a recombinant rat Fc with substitutions in both chains that prevent binding to rFcRn (nonbinding Fc; nbFc) (24) was not observed in either the rFcRn-MDCK or the rFcRn-GFP-MDCK cells. Labeled hdFc, a recombinant rat Fc composed of one wild-type Fc chain and one nbFc chain (24), was also endocytosed by the rFcRn-MDCK cells in a saturable and pH-dependant manner, although at lower levels than wtFc (Figure 2). Taken together, these results show that rFcRn expressed in MDCK cells undergoes proper posttranslational modifications, binds IgG with the expected pH dependence and mediates specific and saturable uptake of Fc ligands at acidic pH.

**Figure 3:**
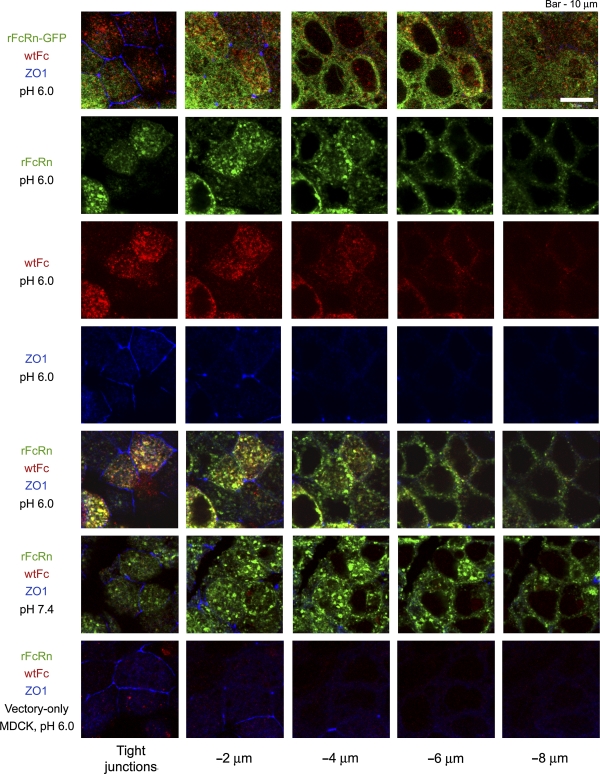
Distribution of internalized wtFc in rFcRn-MDCK, rFcRn-GFP-MDCK and vector-only MDCK cells Filter-grown monolayers were incubated with 1 μM wtFc (recombinant Fc derived from rat IgG2a) for 1 h at pH 6 or pH 7.4 and processed for immunofluorescence using antibodies against wtFc (red) and the tight junction marker ZO-1 (blue). The heavy chain of rFcRn (green) was detected using GFP fluorescence (rFcRn-GFP-MDCK cells) or an antibody against the rFcRn heavy chain (rFcRn-MDCK and vector-only MDCK cells). Optical sections were taken every 2 μm below the level of the tight junctions.

To observe the subcellular localization of endocytosed Fc, the apical surfaces of polarized rFcRn-MDCK, rFcRn-GFP-MDCK or vector-only MDCK cell monolayers were incubated with wtFc (1 μm) at acidic or basic pH and processed for immunofluorescence microscopy. Confocal images (Figure 3) show that internalized wtFc (red fluorescence) is present in rFcRn-positive compartments (green fluorescence) in rFcRn-MDCK and rFcRn-GFP-MDCK cells incubated with ligand at pH 6. The distribution of rFcRn and rFcRn-GFP was not significantly different in the absence of wtFc or IgG compared to when these ligands were present at concentrations ranging from 20 nm to 1 μm (data not shown), contradicting the previous report (25). Also in contrast to the previous study (25), substantially less uptake was observed when the cells were incubated with wtFc at basic pH (pH 7.4) versus at acidic pH (shown for rFcRn-MDCK; Figure 3), indicating that an acidic extracellular environment greatly enhances uptake by allowing Fc to bind rFcRn at the cell surface prior to internalization. The vector-only MDCK cells did not stain with the 1G3 antibody and showed only low levels of internalized wtFc (Figure 3), likely resulting from trace internalization via the fluid phase.

### Bidirectional transcytosis of IgG and Fc

**Figure 4:**
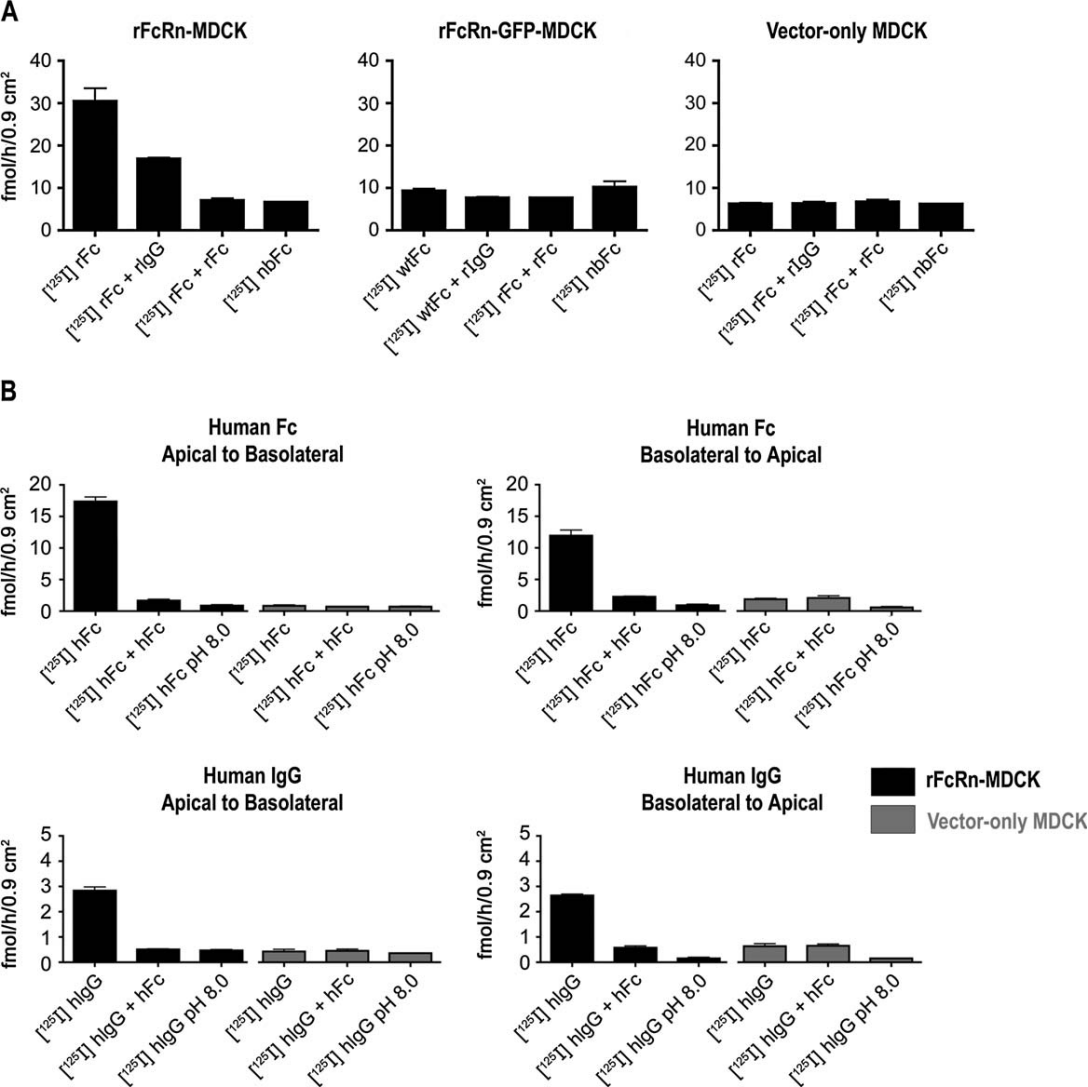
Bidirectional transcytosis of Fc and IgG ligands by rFcRn-MDCK cells Radiolabeled Fc or IgG was added to the loading surface (apical in panel A and apical or basolateral in panel B) of polarized cell monolayers and levels of radioactivity in the media from the nonloading surface were measured after 90 min. Bars represent the mean and standard deviation of triplicate filters. (A) Comparison of apical to basolateral transcytosis of wtFc in three different cell lines. rFcRn-MDCK, rFcRn-GFP-MDCK and vector-only MDCK cells were incubated on the apical surface with 20 nM of [^125^I]wtFc (recombinant Fc derived from rat IgG2a), [^125^I]wtFc plus 500-fold excess unlabeled wtFc or rat IgG (rIgG), or [^125^I]nbFc. (B) Comparison of bidirectional transcytosis of Fc and IgG. rFcRn-MDCK and vector-only MDCK cells were incubated with 20 nM [^125^I]human Fc (hFc) or [^125^I]human IgG (hIgG) on the apical or basolateral surface. Control experiments were performed in the presence of 500-fold excess unlabeled hFc or at pH 8 as indicated.

Radiolabeled rat wtFc (20 nM) was added to the apical surface of the polarized rFcRn-MDCK or rFcRn-GFP-MDCK monolayers at pH 6, while the basolateral surface was maintained at pH 8. The amount of transcytosed ligand in the basolateral reservoir was measured after 90 min. Significantly more wtFc is transported by the rFcRn-MDCK cells than the control MDCK cells (Figure 4A). The presence of a 500-fold excess of competitor (unlabeled rat IgG or wtFc) reduces transcytosis to background levels observed for [^125^I]nbFc.

Surprisingly, rFcRn-GFP-MDCK cells do not mediate specific transport of [^125^I]wtFc (Figure 4A). This lack of detectable transport was observed in at least three other clones of rFcRn-GFP-MDCK cells, and in transcytosis experiments performed in both the apical to basolateral and basolateral to apical directions (data not shown). Because the rFcRn-GFP-MDCK cells do appear to function in a pH-dependent uptake of wtFc (Figure 2), these data suggest that addition of the GFP tag to the rFcRn cytoplasmic tail does not prevent endocytosis, but interferes with postinternalization trafficking events. Further experiments were therefore conducted using only the rFcRn-MDCK cells and vector-only control MDCK cells.

We next compared the ability of rFcRn-MDCK cells to transcytose IgG and Fc ligands in both the apical to basolateral and basolateral to apical directions (Figure 4B). These experiments were conducted using [^125^I]human IgG and [^125^I]human Fc because radiolabeled human IgG is transcytosed more efficiently than radiolabeled versions of other commercially available IgGs (data not shown). We find that both IgG and Fc are transcytosed bidirectionally across the rFcRn-MDCK cells when the loading surface is maintained at pH 6 and the nonloading surface is maintained at pH 8 (Figure 4B). When either ligand is prevented from binding to cell-surface rFcRn by maintaining the loading surface at pH 8, or by saturating surface receptors with unlabeled ligand, we see only background levels of transcytosis. Fc is transported more efficiently than intact IgG in both directions. The lower efficiency of IgG transcytosis as compared with Fc transcytosis may result from steric effects, i.e., the Fab arms present on intact IgG could clash with the membrane when rFcRn is in a ‘standing up’ conformation (11). This is consistent with our observation that intact rat IgG competes less efficiently for endocytosis and transcytosis of [^125^I]wtFc than does wtFc (Figures 2 and 4A).

### Binding, endocytosis and transport studies using rat albumin as an rFcRn ligand

**Figure 5:**
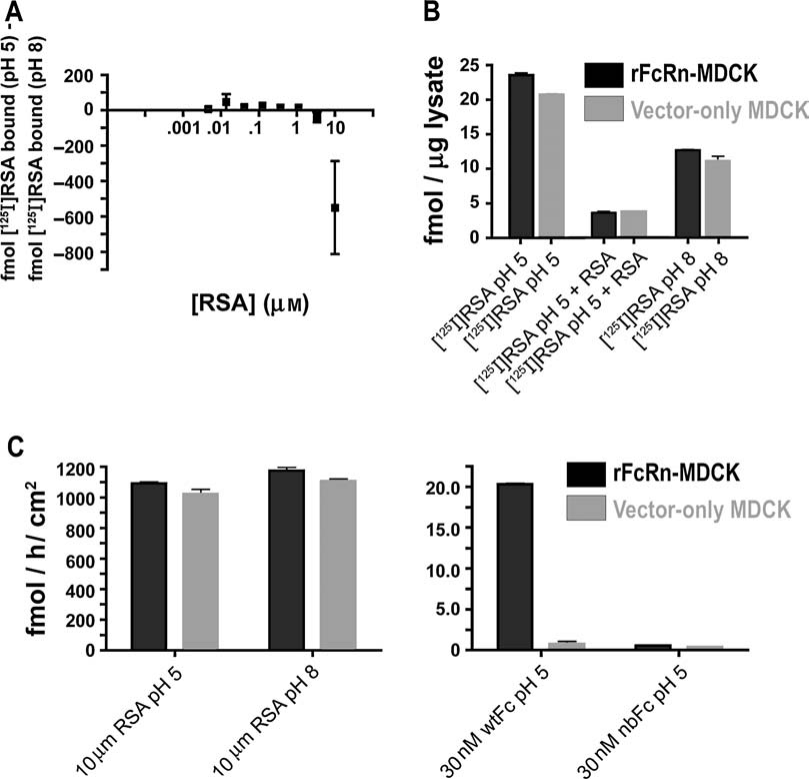
Albumin binding, endocytosis and transport experiments (A) Cell-surface binding. rFcRn-MDCK cells were incubated at pH 5 with [^125^I]RSA at a range of concentrations. For each concentration of labeled RSA, quadruplicate samples were washed at either pH 8 or pH 5 after the incubations, and the radioactivity bound at basic pH was subtracted from the radioactivity bound at acidic pH to determine the amount of specifically bound RSA at each concentration. (B) Endocytosis of RSA. Subconfluent rFcRn-MDCK and vector-only MDCK cells were incubated with 3 μM [^125^I]RSA at pH 5 in the presence or absence of 2 mM unlabeled RSA, or at pH 8, and levels of radioactivity were determined in cell lysates. (C) Apical to basolateral transcytosis of RSA. The apical surfaces of filter-grown cell monolayers were incubated with 10 μM [^125^I]RSA at pH 5 or pH 8 as indicated. Some filters were treated with 30 nM [^125^I]wtFc or [^125^I]nbFc as positive and negative controls, respectively. The levels of radioactivity in the basolateral media were determined after 90 min.

IgG and Fc are both bivalent ligands of FcRn, that is, each contains two potential FcRn-binding sites. To determine whether rFcRn expressed in MDCK cells can act as a functional receptor for a monovalent ligand, rat serum albumin (RSA), we conducted binding, endocytosis and transcytosis assays using [^125^I]RSA. Because RSA has a more acidic pH optimum than IgG for binding to rFcRn (14,27), cell-surface binding assays were performed at pH 5.0. Although rFcRn-MDCK cells incubated at pH 5 specifically bind [^125^I]wtFc (data not shown), cells incubated with [^125^I]RSA and then washed at pH 5 do not bind more RSA than cells washed at pH 8 (Figure 5A); thus, we see no specific binding of RSA to rFcRn-MDCK cells.

We next tested the possibility that binding to cell-surface rFcRn that is undetectable in a binding assay could promote endocytosis of RSA into rFcRn-MDCK cells. A high concentration (3 μM) of [^125^I]RSA was added at pH 5 or 8 to rFcRn-MDCK or to vector-only MDCK cells in the presence or absence of a 100-fold excess of unlabeled RSA. We find that both the rFcRn-MDCK and vector-only cells internalize a high level of labeled RSA at pH 5 and at pH 8, with the greatest amount of internalization being observed when [^125^I]RSA is added at pH 5 without competitor (Figure 5B). Although slightly more RSA is internalized at pH 5 by the rFcRn-MDCK cells than by the vector-only MDCK cells, perhaps representing rFcRn-mediated endocytosis of RSA, the difference is very small compared to the absolute amount of RSA internalized by either cell line.

We also investigated whether the rFcRn-MDCK cells can transcytose RSA. To account for the relatively low affinity of the RSA–rFcRn interaction (14,27), a high concentration of [^125^I]RSA (10 μm) was incubated at the apical surface of rFcRn-MDCK or vector-only-MDCK cells at pH 5 or pH 8. Control experiments using labeled wtFc and nbFc demonstrated that incubation at pH 5 does not disrupt transcytosis of a functional ligand (wtFc) or result in leakage of nbFc across the monolayer (Figure 5C). In the experiments using labeled RSA, we see no evidence of rFcRn-mediated transcytosis. Instead, we observe a high level of transport across both the rFcRn-MDCK and control cells at both acidic and basic pH, with transport being slightly higher for both cell lines at basic pH (Figure 5C). Similar results were obtained in experiments using lower concentrations of [^125^I]RSA (data not shown). The lack of observed transcytosis of RSA in the rFcRn-MDCK cells is consistent with a previous demonstration that little or no albumin is transcytosed across the proximal intestine of neonatal rats under conditions in which 10–35% of the administered IgG is transported into the blood (28).

### Relative affinities of wtFc and hdFc for cell-surface rFcRn

**Figure 6:**
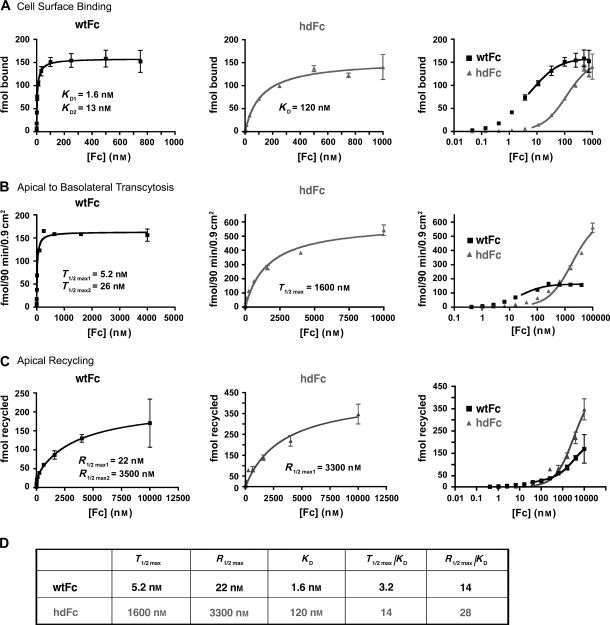
Comparison of binding and transport of wtFc and hdFc by rFcRn-MDCK cells Binding, transcytosis and recycling data are presented separately for wtFc and hdFc (recombinant Fc proteins derived from rat IgG2a) as a linear function of concentration (left and center graphs in panels A–C), and together as a function of the logarithm of the input concentration (right graphs in panels A–C). Data points represent the mean and standard error for quadruplicate (panel A) or triplicate (panels B and C) measurements. The data for wtFc were fit to a two-site binding model, therefore yielding two half-maximal values per graph: half-maximal binding (*K*_D_), half-maximal transport (*T*_1/2max_) or half-maximal recycling (*R*_1/2max_). The data for hdFc were fit to a one-site binding model, yielding one half-maximal value per graph (see *Materials and Methods*). (A) Cell-surface binding of wtFc and hdFc. The amounts of wtFc and hdFc bound to cell-surface rFcRn were determined using ∼500 000 cells at each input concentration. Assuming that each wtFc or hdFc molecule is bound by a single rFcRn molecule at saturating concentrations, there are ∼150 fmol of ligand binding sites in each binding experiment, which corresponds to ∼180 000 surface-accessible sites/cell. (B) Apical to basolateral transcytosis of wtFc and hdFc. (C) Apical recycling of wtFc and hdFc. (D) Comparison of *T*_1/2max_ to *K*_D_ and *R*_1/2max_ to *K*_D_ ratios for wtFc and hdFc. These ratios are predicted to be 1.0 in a perfectly efficient transport or recycling system.

Because we did not observe binding or transyctosis of albumin, a naturally occurring monovalent ligand for FcRn, we used hdFc as a monovalent ligand for comparative studies with wtFc, a bivalent ligand. We first compared the binding of radiolabeled wtFc and hdFc to rFcRn on the surface of rFcRn-MDCK cells. Equilibrium dissociation constants (*K*_D_) were determined by nonlinear regression analysis from plots of bound Fc as a function of Fc concentration (Figure 6A). The wtFc binding data were fit to a two-site binding model assuming two independent classes of binding sites (a high-affinity class, which takes avidity effects into account, and a low-affinity class), yielding *K*_D_ values of 1.6 and 13 nM. The hdFc binding data cannot be fit to a two-site binding model (data not shown), consistent with its single FcRn-binding site. These data were therefore fit to a 1:1 binding model, from which a *K*_D_ of 120 nM was derived.

### Transcytosis and recycling efficiencies of monovalent and bivalent Fc proteins

We next compared the transcytosis and recycling efficiencies of the two forms of rat Fc. First, radiolabeled versions of wtFc and hdFc were added at pH 6 to the apical surface of rFcRn-MDCK monolayers at a range of concentrations. The amount of transcytosed Fc released into the basolateral medium after 90 min was plotted as a function of Fc concentration (Figure 6B), yielding curves that are analogous to the binding isotherms in Figure 6A. At concentrations below 1 μm, wtFc is transported in significantly greater amounts than hdFc. Transcytosis of both proteins is comparable at approximately 1 μm, and for values greater than 1 μm, more hdFc than wtFc is transported to the basolateral media. Nonlinear regression analysis performed on the trancytosis data was used to extract the concentration at which half-maximal transcytosis occurs (*T*_1/2max_), which is analogous to a *K*_D_, the concentration at which half-maximal binding of a ligand to its receptor occurs. As described for the binding data, the wtFc transcytosis data were fit to a two-site model, yielding two *T*_1/2max_ values, 5.2 nM (high-affinity population) and 26 nM (low-affinity population), and the data for hdFc were fit to a one-site model, yielding a single *T*_1/2max_ value (1.6 μm). The fact that the *T*_1/2max_ value for the high-affinity population of receptors that bind wtFc (5.2 nM) is lower than the *T*_1/2max_ value for hdFc (1.6 μm) indicates that bivalency of the Fc ligand contributes to the efficient transcytosis of cargo, particularly at lower ligand concentrations, but that ligand bivalency is not absolutely required for FcRn-mediated transcytosis.

Apical recycling was evaluated by measuring the amount of radioligand returned to the apical media after internalization from the apical surface of rFcRn-MDCK cells. As described for the transcytosis assay, the amounts of recycled wtFc and hdFc were plotted as a function of concentration, and the concentrations at which half-maximal recycling occurs (*R*_1/2max_) were determined from the data by nonlinear regression analyses using a two-site binding model (wtFc) or a one-site binding model (hdFc) (Figure 6C). The *R*_1/2max_ values for recycling of wtFc (*R*_1/2max1_) and hdFc are 22 nM and 3.3 μM, respectively, indicating that bivalency of the Fc ligand also contributes to the efficiency of the recycling process.

To compare the affinities of the two forms of Fc to their transport efficiencies, we calculated the *T*_1/2max_/*K*_D_ and *R*_1/2max_/*K*_D_ ratios for each Fc. For comparisons of wtFc transcytosis and recycling with binding, we are interested in the *K*_D_, *T*_1/2max_ and *R*_1/2max_ values representing the high-affinity population of receptors that are affected by avidity (*K*_D1_, *T*_1/2max1_ and *R*_1/2max_ in Figure 6A–C). The *T*_1/2max1_/*K*_D1_ ratio for wtFc is 3.2, which is significantly smaller than the *T*_1/2max_/*K*_D_ ratio for hdFc (∼14) (Figure 6D); thus, the less efficient transport of hdFc versus wtFc is not fully explained by the differences in affinity. Similarly, the *R*_1/2max1_/*K*_D1_ ratio for wtFc (∼14) is smaller than the *R*_1/2max_/*K*_D_ ratio for hdFc (∼28) (Figure 6D).

Although the *T*_1/2max_ and *R*_1/2max_ values demonstrate that higher concentrations of hdFc than wtFc are required to achieve half-maximal transcytosis or recycling rates, the absolute amounts of hdFc that are transcytosed and recycled are higher than the analogous amounts of wtFc as the concentration of Fc approaches saturation (values above ∼1 μM) (Figure 6B,C). The reason for the higher levels of hdFc transport is unclear. It may result from the fact that a given number of receptors can transport twice as many hdFc molecules as wtFc because hdFc can be bound and transported by only one receptor, whereas wtFc can be bound and transported by two receptors. In addition, allowing each receptor to act as an independent trafficking unit may increase the overall rate at which individual receptors can complete one full round of ligand internalization and transport as compared to when the receptors are required to transport cargo in sets of two.

### Comparison of localization of wtFc and hdFc in rFcRn-MDCK cells

To address whether the increased efficiency of wtFc over hdFc transport results from different intracellular fates of the two forms of Fc, we compared the intracellular localizations of internalized wtFc and hdFc with a marker for early endosomes (EEA1) and with a marker for lysosomes (the lysosomal membrane protein LAMP-2).

**Figure 7:**
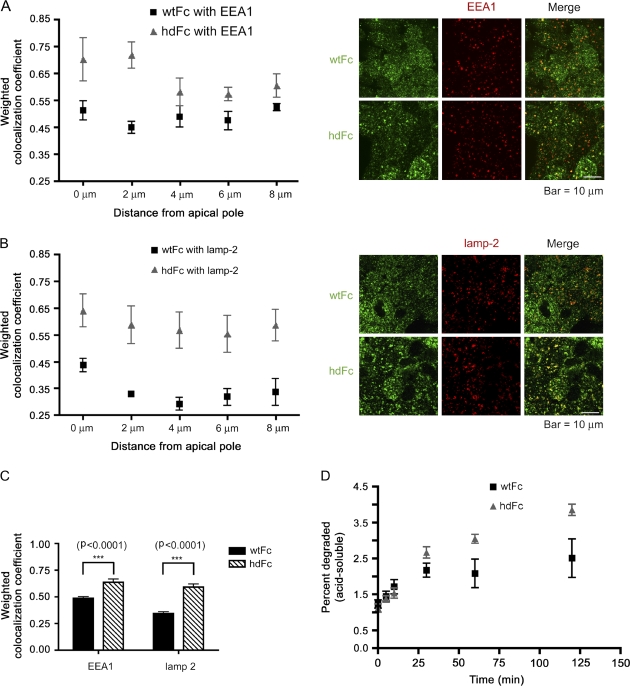
Comparison of intracellular localization and degradation of wtFc and hdFc (A and B) The apical surface of rFcRn-MDCK cells was incubated with 1 μM wtFc or hdFc (recombinant Fcs derived from rat IgG2a) at pH 6 for 1 h, then fixed and processed for immunofluorescence using labeled antibodies against rat Fc and specific markers. The degree of colocalization of the Fcs with EEA1 (panel A) or LAMP-2 (panel B) is shown as a function of distance from the apical pole on the left of each panel (*n* = 3 or 4 for each data point). The percentage of above-threshold Fc pixels colocalizing with above-threshold EEA1 or LAMP-2 pixels was determined in each optical section as described in the *Materials and Methods*. Confocal images of the distributions of wtFc or hdFc (green) and EEA1 (red) (panel A) or wtFc or hdFc (green) and canine LAMP-2 (red) (panel B) are shown for a subapical optical section on the right of each panel. Regions of colocalization appear yellow in the merged images. (C) Averages of the colocalization coefficients for five optical sections spanning the whole cell. Significantly more (p < 0.0001) hdFc than wtFc colocalizes with EEA1 (*n* = 15 and *n* = 19, respectively) and with LAMP-2 (*n* = 20). (D) Comparison of degradation of wtFc and hdFc. [^125^I]labeled wtFc or hdFc was internalized at pH 6 from the apical surface of rFcRn-MDCK cells for 1 h, after which the cells were washed at pH 8. The cells were returned to 37°C for the indicated time points, after which acid-soluble radioactivity in the media was measured. The percent of each Fc that was degraded was determined as described in the *Materials and Methods*.

After 1 h of internalization from the apical surface, both wtFc and hdFc are present in discrete compartments throughout the cell (Figure 7A,B), including EEA1-positive compartments (Figure 7A), demonstrating that rFcRn-bound Fc proteins enter the endosomal network after being endocytosed. Although the mean intensity of the wtFc and hdFc staining is similar (see *Materials and Methods*), more hdFc than wtFc is colocalized with EEA1, as demonstrated by quantitative analysis of the confocal image data to determine the percent colocalization of above-threshold fluorescence for each type of Fc with EEA1 (see *Materials and Methods*). The degree of hdFc colocalization with EEA1 is consistently higher throughout the cell than the colocalization of wtFc with EEA1, with the most significant differences observed in the apical sections of the monolayer (left, Figure 7A). Averaged over the entire cell, 64 ± 3.0% of the hdFc fluorescence colocalizes with EEA1-positive compartments compared with 49 ± 1.4% of the wtFc fluorescence (Figure 7C). A more striking difference is observed for colocalization of the two Fcs with the lysosomal marker LAMP-2 (Figure 7B), in which 59 ± 2.8% of hdFc fluorescence colocalizes with LAMP-2-positive compartments compared with 34 ± 1.7% of wtFc fluorescence (Figure 7C).

The observation that more hdFc than wtFc is present in LAMP-2-positive compartments suggests that more hdFc than wtFc is degraded following internalization. To test this possibility, we measured the amounts of degraded Fc (acid-soluble radioactivity) released as a function of time by cells incubated with 1 μm wtFc or hdFc. As shown in Figure 7D, more degraded radioligand is released from hdFc-treated cells than from wtFc-treated cells. This difference is significant for postincubation times of 30 min or more, confirming that a substantially greater fraction of a single cohort of internalized hdFc is degraded compared to wtFc.

## Discussion

Ligand valency is a critical feature of many receptor–ligand interactions. While many receptors that mediate intracellular signal transduction bind bivalent ligands to form 2:1 receptor:ligand complexes (e.g., platelet-derived growth factor receptor, colony-stimulating factor 1 receptor and stem cell factor receptor) [reviewed by Heldin (29)], receptors involved in ligand transcytosis and transport more commonly bind monovalent ligands to form 1:1 complexes (e.g., pIgR, low-density lipoprotein receptor and transferrin receptor) [reviewed by Tuma and Hubbard (30)]. FcRn was first identified as a receptor for IgG, a bivalent ligand, and the crystal structure of rFcRn in complex with wtFc showed that two FcRn molecules can bind simultaneously to a dimeric Fc (11). Studies of purified proteins in solution confirmed a 2:1 FcRn:Fc stoichiometry (24,31–33), and *in vivo* studies in mice showed that both FcRn-binding sites on Fc are required for efficient transcytosis and protection from catabolism (12,13). However, the discovery that FcRn also serves as a protection receptor for serum albumin (14,34), a monovalent ligand, suggests that ligand bivalency is not a general requirement for FcRn-mediated ligand trafficking.

Here we investigate the effects of valency on FcRn-mediated ligand trafficking to determine whether bivalency of an FcRn ligand is a strict requirement for proper trafficking, or if it merely acts to increase the efficiency of the trafficking process. In the former case, one would expect the binding of the Fc or IgG ligand by two FcRn molecules to act as a signal, perhaps by bringing the receptors into close spatial proximity to facilitate interactions with cytosolic trafficking components. In the latter case, the binding of each ligand by two receptors would facilitate transport through an avidity effect, ensuring that the ligand molecule, once bound and internalized, remains in complex with its receptor throughout the transport process.

To evaluate the effects of ligand valency on FcRn-mediated transport, we generated stable MDCK cell lines expressing rFcRn that can be used as a model system for studies of FcRn-mediated transport of IgG and Fc ligands. Two cell lines were generated: rFcRn-MDCK, which expresses full-length rFcRn and the rFcRn light chain rβ_2_m, and rFcRn-GFP-MDCK, which expresses an rFcRn-GFP chimeric protein together with rβ_2_m. The expressed rFcRn proteins both bind to IgG at acidic but not at basic pH (Figure 1B), the characteristic pH dependency of the FcRn–IgG interaction (3). Functional binding and internalization of Fc ligands were demonstrated in polarized rFcRn-MDCK and rFcRn-GFP-MDCK monolayers at acidic pH (Figure 2). We observe only background amounts of wtFc internalized at pH 8, suggesting that fluid-phase uptake at basic pH is not significant at the relatively low concentration (20 nM) used for these experiments. Thus, an acidic extracellular environment greatly enhances the efficiency of ligand uptake, most likely by allowing IgG or Fc to bind rFcRn molecules transiently exposed to the cell surface prior to reinternalization. However, confocal analyses of rFcRn-MDCK cells that have internalized Fc ligands at a significantly higher concentration (1 μM) indicate that a low, but detectable, amount of wtFc is internalized at pH 7.4 (Figure 3), consistent with the assumption that non-receptor-mediated uptake of IgG at basic pH occurs in FcRn-mediated placental transport and in FcRn-mediated recycling of IgG by vascular endothelial cells (35). In these *in vivo* situations, the high concentration of IgG in the blood (50–100 μM) (36) likely facilitates fluid-phase uptake of IgG.

The rFcRn-MDCK cells transport IgG and Fc in both the apical to basolateral and basolateral to apical directions when the loading surface is incubated at acidic pH (Figure 4) as previously observed for hFcRn-transfected MDCK cells (16–18), rFcRn-transfected IMCD cells (20–22), and rat alveolar cells (23). Interestingly, in contrast to the studies on hFcRn in MDCK cells, in which higher levels of transcytosis occurred in the basolateral to apical direction (16,17), rFcRn expressed in IMCD and rat alveolar cells transports more ligand in the apical to basolateral direction (20,23). Consistent with these results, the rFcRn-MDCK cells described here transport more Fc in the apical to basolateral direction and roughly equal amounts of IgG bidirectionally (Figure 4), suggesting fundamental differences in the preferred directionality of ligand transport by the rat and human receptors.

Although the rFcRn-MDCK cells function in transcytosis of IgG and Fc ligands, we do not observe significant levels of wtFc transport in the rFcRn-GFP-MDCK cells (Figure 4A). The most likely explanation is that the addition of GFP to the C-terminus of the rFcRn cytoplasmic tail interferes with the binding of downstream effectors responsible for mediating intracellular trafficking processes. This is surprising, as a C-terminal GFP fusion of hFcRn has been shown to function in recycling of IgG ligands in the human endothelial cell line HMEC-1.CDC (35,37). Whether this discrepancy is due to differences in the interactions required for transcytosis versus recycling, differences in the behavior of the receptors when expressed in HMEC-1.CDC versus MDCK cells or fundamental differences between the hFcRn and rFcRn proteins is not clear. However, the fact that rFcRn-GFP-MDCK cells function in specific uptake of Fc ligands (Figure 2) suggests that signals responsible for endocytosis are intact, implying different signaling mechanisms for endocytosis versus transcytosis, or that rFcRn can internalize bound ligand via non-specific membrane flow after binding to ligand molecules at the cell surface.

Having demonstrated that rFcRn expressed in MDCK cells functions in binding, endocytosis and transcytosis of Fc and IgG ligands, both of which are naturally bivalent, we sought to study the interaction of rFcRn with RSA, a monovalent ligand that binds to rFcRn with the same pH dependency as the FcRn–IgG interaction and for which FcRn acts as a protection receptor *in vivo*(14,34). Despite using high ligand concentrations (10 μM) and low pH (pH 5) to facilitate binding (14,27), we were not able to detect specific binding of radiolabeled RSA to rFcRn (Figure 5A). We note, however, that although the experiments were performed at concentrations near or above the *K*_D_ of hFcRn for albumin (∼1–5 μM) (27), we could not perform experiments at the concentration of albumin in serum (∼270 μm) (38) because of background problems. When incubated at 3 μm, rFcRn-MDCK cells did not internalize significantly more RSA than control cells that do not express rFcRn (Figure 5B). However, both cell lines internalized less labeled RSA in the presence of competing unlabeled RSA, suggesting that much of the observed internalization was due to non-rFcRn-dependent binding of the labeled RSA to constituents of the cell surface. In addition, the levels of apical to basolateral transport of labeled RSA were similar in rFcRn-expressing and control cell lines, and the degree of transport was not significantly altered in either cell line by incubating the loading surface at pH 8 rather than at pH 5 (Figure 5C). Taken together, these results suggest that RSA binds non-specifically to components present on the surface of MDCK cells; thus, these cells are not an appropriate system in which to study the interaction of rFcRn with RSA.

To investigate the potential requirement for two FcRn-binding sites on an FcRn ligand, we compared the ability of rFcRn-MDCK cells to transcytose and recycle monovalent and bivalent forms of rat Fc (hdFc and wtFc, which contain one and two FcRn-binding sites, respectively) (Figure 6B,C). The experiments were performed over a wide range of concentrations (0.15 nM–10 μM) to compensate for avidity affects that favor binding of wtFc over hdFc at low concentrations. The results revealed that hdFc is specifically transcytosed and recycled by rFcRn, but that more wtFc than hdFc is transported at low concentrations (<1 μM). Previous studies suggesting that two FcRn-binding sites on mouse Fc are required for FcRn-mediated rescue from catabolism (13) and transcytosis across the intestine of neonatal mice (12) are consistent with the present results if the Fc concentrations used in the *in vivo* studies fell within the range in which wtFc is more efficiently transported by FcRn than hdFc. The present study reveals that the amount of wtFc transcytosed reaches a relatively constant level at concentrations above 150 nM, whereas the amount of hdFc transcytosed continues to increase with concentration, such that more hdFc than wtFc is transcytosed at concentrations above 1 μM (Figure 6B). At a concentration of 10 μM, at least twice as much hdFc is transcytosed or recycled as wtFc. This suggests that two rFcRn molecules are bound to each wtFc during the transport process, whereas only one rFcRn molecule is bound to each hdFc, resulting in twice as many available receptors to transport the monovalent hdFc as there are for the transport of the bivalent wtFc.

To determine how the differences in rFcRn-mediated transport of wtFc versus hdFc are related to the apparent affinity of rFcRn for each ligand, we compared the concentrations of each protein that result in half-maximal binding to cell-surface rFcRn (*K*_D_) with the concentrations that result in half-maximal transcytosis across a polarized monolayer (*T*_1/2max_) and with the concentrations that result in half-maximal recycling of ligand back to the apical surface (*R*_1/2max_). In a perfectly efficient transcytosis system, each binding event would result in a single transcytosis event, and *T*_1/2max_ would equal *K*_D_. In the present study, we observe that the *T*_1/2max_/*K*_D_ ratio for wtFc transcytosis is ∼3, whereas the ratio for hdFc transcytosis is ∼14 (Figure 6D). For the recycling experiments, the *R*_1/2max_/*K*_D_ ratio for wtFc is ∼14, whereas the ratio for hdFc is ∼28. Thus, a greater number of individual binding events result in successful transcytosis or recycling of the bivalent wtFc, whereas transcytosis or recycling of the monovalent hdFc requires the input of more ligand to achieve saturation of the transport system. Interestingly, the *T*_1/2max_/*K*_D_ and *R*_1/2max_/*K*_D_ values suggest that a given Fc molecule, once bound to FcRn at the apical surface, is more likely to be transcytosed than recycled.

The observed differences in the relative efficiencies of transport of the bivalent and monovalent Fcs could be related to differences in the subcellular fates of the two molecules during rFcRn-mediated trafficking. For example, a higher proportion of endocytosed hdFc molecules may enter a degradative pathway, whereas a higher proportion of wtFc molecules are transcytosed or recycled. Consistent with this hypothesis, we observed a greater amount of hdFc in both lysosomal (LAMP-2 positive) and early endosomal (EEA1 positive) compartments as compared to wtFc (Figure 7), suggesting that more wtFc than hdFc is transported to compartments downstream of the early endosomes and that less wtFc than hdFc is sent to degradative compartments. The quantitative colocalization experiments were conducted using a ligand concentration of 1 μm, a concentration at which roughly equal amounts of wtFc and hdFc are bound and transported by rFcRn-expressing MDCK cells (Figure 6A–C). Under these conditions, we presume that all wtFc or hdFc molecules on the cell surface are initially bound by a single rFcRn molecule prior to internalization and that the amount of each ligand entering the cell is approximately equal. Consistent with this assumption, digital image analysis shows that the average mean pixel intensities for Fc fluorescence in all images used in these experiments are similar (see *Materials and Methods*). Therefore, the higher proportion of hdFc seen in LAMP-2- and EEA1-positive structures is not the result of greater absolute amounts of hdFc entering into the cell. Instead, the data are consistent with a model whereby equal amounts of both wtFc and hdFc initially enter the cell but, subsequent to internalization and delivery to EEA1-positive early endosomes, a greater proportion of wtFc than hdFc molecules remain bound to FcRn and are transported to downstream compartments involved in transcytosis and/or recycling. Conversely, a greater proportion of hdFc molecules remain in early endosomes prior to being transferred to the lysosomal degradation pathway along with other fluid-phase components. This model is also supported by our demonstration that, following internalization of a single cohort of wtFc or hdFc ligand, a higher percentage of hdFc is released from rFcRn-MDCK cells as degraded (acid-soluble) material (Figure 7D).

The observation that an Fc ligand containing only one FcRn-binding site is preferentially trafficked to lysosomes and degraded compared with a bivalent Fc ligand can be explained by an avidity effect; because the hdFc cannot be cross-linked by adjacent FcRn molecules, it is predicted to dissociate more readily from FcRn inside endosomes, and unbound hdFc could then enter a default degradative pathway with other molecules in the fluid phase. The present demonstration that FcRn-mediated transcytosis and recycling of hdFc occur, although at a lower efficiency than transcytosis and recycling of wtFc, does not address whether an oligomeric structure of FcRn dimers linked by homodimeric Fc or IgG molecules (10,11) is present inside endosomes, but rules out that it is required for proper transport of vesicles containing FcRn–IgG or FcRn–Fc complexes.

## Materials and Methods

### Construction of expression vectors

The gene encoding rFcRn was modified by polymerase chain reaction (PCR) to incorporate 5′*Asp*718 and 3′*Hin*dIII restriction sites and subsequently subcloned with an *Asp*718/*Hin*dIII double digest into the mammalian cell expression vector pCB6-*Hin*dIII (kindly provided by Ira Mellman, Yale University), which carries a neomycin resistance gene for G418 selection. To make the FcRn–GFP fusion construct, the enhanced GFP (EGFP) gene was amplified from the pEGFP-1 vector (Clontech, Mountain View, CA, USA) via PCR to remove the start codon and to introduce a 5′ in-frame *Xho*I site and a 3′*Hin*dIII site and subcloned into the pBluescript II SK− vector (Stratagene, La Jolla, CA, USA). Polymerase chain reaction was used to introduce a 5′*Asp*718 site and an in-frame 3′*Xho*I site at the 3′ end of the rFcRn gene, which was then subcloned into the EGFP Bluescript vector. The resulting open reading frame, which encoded the entire rFcRn amino acid sequence, a leucine-glutamate linker region and EGFP without its N-terminal methionine, was subcloned with a *Asp*718/*Hin*dIII double digest into the expression vector pCB6-*Hin*dIII. We used a previously described rβ_2_m expression vector (39), which does not contain a selectable marker, to coexpress rβ_2_m along with full-length rFcRn and the rFcRn-GFP chimeric protein.

### Antibodies

The mouse monoclonal antibodies 1G3 (anti-rFcRn heavy chain) and 4C9 (anti-rβ_2_m) were generated in our laboratory (40) and can be purchased from the American Tissue Culture Collection. 2B10C11, a mouse monoclonal antibody against rβ_2_m, was the kind gift of Lennart Lögdberg. 1G3, 4C9 and 2B10C11 were directly conjugated to AlexaFluor488-NHS, AlexaFluor546-NHS or AlexaFluor633-NHS (Molecular Probes, Carlsbad, CA, USA) according to the manufacturer’s protocol and used for immunofluorescence (1G3 and 4C9) and flow cytometry (2B10C11) experiments. Labeled antibodies were separated from unconjugated dye using 10 000-kDa cutoff dextran desalting columns (Pierce, Rockford, IL, USA), and the concentration and degree of labeling were determined spectrophotometrically using an extinction coefficient at 280 nm of 202 000 m^−1^ cm^−1^ for the antibodies. A polyclonal rabbit antiserum used for Western blotting was raised against purified rFcRn/rβ_2_m heterodimers. Polyclonal rabbit anti-ZO1 was from Zymed (San Francisco, CA, USA). Mouse monoclonal anti-EEA1 was from BD Transduction Labs (San Jose, CA, USA), and the mouse monoclonal antibody AC17 against the canine lysosomal membrane protein lamp-2 (41,42) was a kind gift from Dr E. Rodriguez-Boulan (Weill Medical College, Cornell University). AlexaFluor488-, 546-, and 647-labeled secondary antibodies (goat anti-mouse, goat anti-rabbit and goat anti-rat) were purchased from Molecular Probes.

### Maintenance and transfection of cell lines

MDCK type II cells (generously provided by Keith Mostov, UCSF) were maintained in MEM (GibcoBRL, Carlsbad, CA, USA) supplemented with 10% FBS (HyClone, Logan, UT, USA) at 37°C, 5% CO_2_. Cells were fed every other day and passaged once weekly. MDCK II cells were cotransfected with expression vectors encoding full-length rFcRn or rFcRn-GFP and rβ_2_m using Lipofectamine 2000 (Invitrogen, Carlsbad, CA, USA) and selected with 0.5 mg/mL G418 (Invitrogen). Resistant colonies were tested for uptake of fluorescently labeled rat Fc at pH 6.0. Colonies from each transfection were picked with cloning cylinders and expanded for further analysis. Colonies were analyzed by flow cytometry for binding to Alexa488-conjugated 1G3 (anti-rFcRn) (40) and/or Alexa633-conjugated 2B10C11 (anti-rβ_2_m). At least three clones from each transfection were assayed by immunofluorescence for expression of both the heavy and light chains of rFcRn as well as for the ability to endocytose and transcytose radiolabeled rat Fc at acidic pH. We also generated MDCK II cells transfected with the pCB6H vector lacking an inserted gene to serve as a negative control (vector-only MDCK cells). All cell lines were maintained under constant drug selection.

The integrity of filter-grown, cell-transfected MDCK monolayers was evaluated by seeding cells at superconfluent density on filter supports and recording daily measurements of the TEER. The TEER typically plateaued after 3 or 4 days at ∼250–300 Ωcm^2^, a characteristic range for polarized MDCK II cells (43).

For experiments requiring polarized cell monolayers, cells were seeded at superconfluent density (1 × 10^6^ cells/mL) onto 12-mm Transwell polyester filters (Corning Costar, Acton, MA, USA), with 0.5 and 1.5 mL of media in the apical and basolateral reservoirs, respectively. Cells were fed daily beginning 2 days after initial seeding and used for experiments on the fourth or fifth day postplating.

### Western blot and IgG binding analyses

Western blot analyses to evaluate FcRn expression were performed after denaturing lysis of transfected cells by boiling in 0.5% SDS and vortexing. After centrifuging to remove insoluble material, samples of each lysate [10 μg of total protein as determined by bicinchoninic acid (BCA) assay from Pierce] were loaded onto a 15% SDS polyacrylamide gel and run under reducing conditions. Proteins were transferred to nitrocellulose, probed with a 1:2000 dilution of a polyclonal rabbit antiserum against the rFcRn ectodomain and then treated with peroxidase-conjugated goat anti-mouse secondary antibody (Jackson Immunoresearch Laboratories, West Grove, PA, USA) and detected by enhanced chemiluminescence (Amersham, Piscataway, NJ, USA).

For evaluations of IgG binding, cells were lysed in 5 mg/mL 3-[(3-Cholamidopropyl)dimethylammonia]-1-propanesulfonate (CHAPS), 130 mM NaCl, 20 μM ethylenediaminetetraacetic acid (EDTA), pH 5.9 (15 mM 2-(N-Morpholino)ethanesulfonic acid [MES]) or pH 8.0 (15 mM 4-(2-Hydroxyethyl)piperazine-1-ethanesulfonic acid [HEPES]), supplemented with a protease inhibitor cocktail for mammalian cells (Sigma, St Louis, MO, USA). After centrifuging to remove insoluble material, lysates were incubated with 50 μL human IgG-Sepharose (Amersham) pre-equilibrated to pH 5.9 or pH 8.0 in lysis buffer overnight at 4°C. The complexes were washed four times with 1 mg/mL CHAPS, 30 mM NaCl, 20 μM EDTA, pH 5.9 or 8.0. Bound proteins were eluted by boiling in double-strength sample buffer and processed for SDS-PAGE and Western blotting to detect rFcRn as described above.

### Preparation of FcRn ligands for uptake and transport assays

Rat FcRn binds all four subclasses of rat, human and mouse IgGs with roughly equal affinities (44); therefore, IgGs and Fcs from these three species were used for different experiments based on availability or convenience.

Three forms of rat Fc, wtFc, hdFc and nbFc, were expressed in Chinese hamster ovary (CHO) cells and purified as previously described (45). The Fc-expressing CHO cell line was generated by transfection of expression vectors encoding wtFc (rat IgG2a residues 223–447) and nbFc (IgG2a residues 223–447 with mutations that disrupt FcRn binding (Thr-252 to Gly, Ile-253 to Gly, Thr-254 to Gly, His-310 to Glu, His-433 to Glu and His-435 to Glu) and a C-terminal factor Xa cleavage site and 6x-His tag). The hdFc and nbFc were purified from supernatants of CHO cells secreting a mixture of wtFc, hdFc and nbFc as described (24). Briefly, CHO supernatants were first passed over a nickel-nitrilotriacetic acid (Ni-NTA) column, allowing separation of wtFc from 6x-His tagged species. The hdFc and nbFc were eluted from the Ni-NTA column, then passed over an FcRn affinity column at pH 6.0. The hdFc was eluted from the FcRn column at pH 8.0, and nbFc was recovered from the flowthrough.

Human Fc and rat and human IgGs, each a mixture of the four IgG subclasses, were purchased from Jackson Immunoresearch Laboratories. Purified samples of the monoclonal antibodies 12C7 (anti-*Drosophila* methuselah) and 1C5 (anti-Zn-α2-glycoprotein) (46) were used as mouse IgG1 proteins. Rat serum albumin was purchased from Sigma. All proteins were passed over a Superdex 200 gel filtration column (Amersham) to remove oligomeric species prior to experiments.

Human IgG, rat IgG, mouse IgG1 monoclonal antibodies, human Fc, the recombinant rat Fcs (wtFc, hdFc and nbFc) and RSA were iodinated to a specific activity of 2–5 μCi/μg using Na[^125^I] (MP Biomedicals, Irvine, CA, USA) and IODO-GEN (Pierce). Radiolabeled ligands were buffer exchanged into HEPES buffered saline (25 mM HEPES, 150 mM NaCl, pH 7.4) by two subsequent passages over Bio-Spin 30 prepacked acrylamide columns (30 000 molecular weight cutoff) (BioRad, Hercules, CA, USA). Protein concentrations were determined by BCA assay (Pierce) using bovine serum albumin or bovine γ-globulin as standards.

### Quantitative endocytosis assay

Cells were grown in 12-well tissue culture plates until ∼80–90% confluent. Prior to incubations with ligand, the cells were serum starved for 20–30 min in Hanks’ balanced salt solution, 1.3 mM CaCl_2_, 0.8 mM MgSO_4_ (HBSS+) adjusted to either pH 5.9 or pH 8.0 with MES or HEPES (10 mM each), respectively. [^125^I]wtFc was added to a final concentration of 20 nM in HBSS+ pH 5.9, and plates were incubated in a 37°C circulating water bath for the indicated amounts of time. For competitor studies, 10 μm unlabeled wtFc or rat IgG was added during the preincubation. The cells were cooled on ice, washed four times with ice-cold HBSS+ pH 8.0 to remove surface-bound ligand and then lysed in 0.1 N NaOH. The radioactivity present in the lysates was counted on a Beckman 5500 γ-counter and converted to fmoles of protein using the specific activity of the radiolabeled ligand.

Quantitative endocytosis assays involving [^125^I]RSA were done as described above, except that the concentrations of [^125^I]RSA and unlabeled RSA were 3 μm and 2 mM, respectively, and the incubations were performed at pH 5.0.

### Cell-surface binding assay

For assays of Fc binding to cell-surface FcRn, subconfluent rFcRn-MDCK cells were lifted from plates by incubating in calcium/magnesium-free HBSS and 4 mM EDTA. Cells were washed twice in binding buffer (HBSS+/1% ovalbumin/1 mM KI/10 mM MES, pH 5.9), and triplicate samples of ∼500 000 cells were incubated with serial threefold dilutions of radiolabeled Fc (0.4–900 nm) in a 100 μL volume for 1 h at 4°C with shaking. The binding reactions were collected onto a 96-well filter plate (Durapore membrane, 0.65- μm pore size) (Millipore, Billerica, MA, USA) by centrifugation at 1500 × *g* for 1 min. The filter wells were washed four times with 250 μL of binding buffer at pH 5.9, and the filter circles were punched out into glass vials and counted for radioactivity. A duplicate set of binding reactions performed in parallel were washed four times with HBSS+/1% ovalbumin/1 mM KI/10 mM HEPES, pH 8.0, to serve as a control for non-specific binding. The amount of radioactivity bound to samples washed at pH 8 was subtracted from the corresponding values for samples washed at acidic pH to yield values for specific binding of the radiolabeled ligand.

The RSA binding assays were conducted as described above, except that the binding reactions were performed at pH 5.0 and washed at pH 5.0 or 8.0 (control experiments), and the concentration of [^125^I]RSA ranged from 4 nM to 10 μM.

### Radioligand transcytosis and recycling assays

For transport assays involving only Fc (Figure 4A), we used radiolabeled rat wtFc. For assays comparing IgG and Fc transport, we used radiolabeled human IgG and human Fc (Figure 4B) because radiolabeled human IgG was transported more efficiently than radiolabeled versions of either rat IgG or two different mouse monoclonal antibodies (data not shown). Human IgG binds as well as rat IgG to rFcRn (44).

For transcytosis experiments involving IgG and Fc ligands, polarized cell monolayers were washed once with HBSS+/1% ovalbumin/1 mM KI buffered to pH 5.9 (loading surface) or pH 8.0 (nonloading surface or loading surface for pH 8 control experiments). Filters were preincubated for 20 min with HBSS+ pH 5.9 on the loading surface (or pH 8.0 for control experiments) and HBSS+ pH 8.0 on the nonloading surface. For competition experiments, the unlabeled competing ligand was present in the loading surface medium during the preincubation step. [^125^I]Fc (rat or human) or [^125^I]IgG (rat or human) was added directly to the loading media to a final concentration of 20 nM at pH 5.9 or 8.0. Plates were incubated for 90 min in a 37°C circulating water bath, after which media from the nonloading surface were collected and precipitated at 4°C with 10% (vol/vol) trichloroacidic acid (TCA). The TCA-insoluble (intact ligand) fractions were counted on a Beckman 5500 γ-counter. For experiments comparing the relative transport efficiencies of [^125^I]wtFc, -hdFc or -nbFc, the radiolabeled proteins were added at concentrations ranging from 0.15 nM to 10 μM. The amount of [^125^I]nbFc that moved across the monolayers for a given concentration was assumed to represent non-specific transport or leakage across the monolayer and was subtracted from the corresponding values for wtFc and hdFc to yield values for specific rFcRn-mediated transport.

Quantitative transcytosis assays involving [^125^I]RSA were done as described above, except that the concentration of [^125^I]RSA was 10 μm, and the apical surface was maintained at pH 5.0 or at pH 8.0 (control experiments). Filters incubated with 30 nM [^125^I]nbFc at pH 5.0 served as a control for the integrity of the monolayer, and filters incubated with 30 nM [^125^I]wtFc at pH 5.0 served as a positive control for the ability of the cells to function in FcRn-mediated transcytosis at pH 5.0.

For apical recycling experiments involving labeled Fcs, filter-grown monolayers were washed and preincubated as described above for the IgG and Fc transcytosis assays. [^125^I]labeled wtFc, hdFc or nbFc was added to the apical surface at pH 5.9 at concentrations ranging from 0.15 nM to 10 μM, while the basolateral surface was maintained at pH 8.0. After a 30-min incubation at 37°C, the cells were cooled on ice and washed rapidly five times with ice-cold HBSS+ pH 8.0. Prewarmed HBSS+ pH 8.0 was then added to the apical and basolateral surfaces. After a 60-min incubation at 37°C, media from the apical surface were collected and TCA precipitated, and the TCA-insoluble fractions were counted as described above.

### Analyses of data for binding and transport assays

The amount of specifically bound or transported Fc was plotted as a function of concentration, and nonlinear regression analyses of the data were performed using GraphPad Prism version 4.0b for Macintosh (GraphPad Software, San Diego, CA, USA). As previously described for biosensor-based binding data (24), half-maximal binding (*K*_D_), transcytosis (*T*_1/2max_) or recycling (*R*_1/2max_) values were derived by fitting the wtFc data to a two-site model, which assumes two independent classes of receptors (a high-affinity class and a low-affinity class), and the hdFc data to a one-site model.

### Preparation of samples for immunofluorescence experiments

Cells were grown on permeable filter supports for 3–4 days to allow formation of a polarized monolayer. The filters were preincubated in HBSS+ pH 8.0 for 20–30 min. The loading surface was then washed with HBSS+ pH 5.9, and ligands were added to the loading surface at the indicated concentrations in the same buffer. The nonloading surface was maintained in HBSS+ pH 7.4 throughout the experiment. Filter plates were incubated at 37°C in a circulating water bath for the indicated times after which the cells were placed on ice and processed for immunofluorescence.

For immunofluorescence staining, cells were washed briefly with ice-cold PBS+ (PBS supplemented with 1 mM CaCl_2_, 0.5 mM MgCl_2_ and 0.25 mM MgSO_4_) and fixed using a pH-shift protocol (47). Briefly, cells were incubated for 5 min at room temperature in 4% paraformaldehyde (PFA) in 80 mM 1,4-piperazinebis-(ethanesulphonic acid) (PIPES), pH 6.5, 5 mM EGTA and 2.0 mM MgCl_2_, and then transferred to 4% PFA, 100 mM sodium borate, pH 11.0 for 10 min. After quenching excess aldehyde with freshly prepared 75 mM NH_4_Cl and 20 mM glycine in PBS for 10 min, the cells were washed twice with PBS and blocked for 30 min at room temperature in PBS containing 8% normal goat serum and 0.025% saponin. Cells were then incubated overnight at 4°C in blocking buffer containing one or more of the following primary antibodies: fluorescently conjugated 1G3 or 4C9 (5 μg/mL), mouse anti-EEA1 (1:100 dilution), rabbit anti-ZO1 (1:25 dilution) or AC17 (1:500). Cells were washed and then incubated with the appropriate AlexaFluor-conjugated secondary antibodies diluted 1:500 in blocking buffer for 1 h at room temperature. For experiments involving localization of internalized Fc proteins, unlabeled ligands were detected after internalization using AlexaFluor-conjugated goat anti-rat IgG antibodies. After extensive washing with PBS, cells were treated with 0.1% Triton X-100 for 5 min, washed once with PBS and then postfixed with 4% PFA in 100 mM sodium cacodylate, pH 7.4 for 30 min at room temperature. The cells were washed twice more with PBS, and the filters were cut out of their holders using a scalpel and a fine-tipped pair of forceps. Excised filters were mounted on glass slides using ProLong Gold antifade medium (Molecular Probes), sealed with beeswax and stored at −20°C until viewing on a confocal microscope.

### Confocal microscopy

All experiments were conducted using an inverted Zeiss LSM META confocal microscope equipped with a Zeiss Plan-Apochromat 100× oil objective (numerical aperture 1.4). Green fluorophores were excited with the 488-nm line of an argon ion laser. Orange and far-red dyes were excited with the 543 and 633- nm lines of a He-Ne laser, respectively. Fluorescence was detected through a variable confocal pinhole set to 1.0 Airy units for the longest wavelength used within a given experiment, and the pinholes of other channels were adjusted to maintain a constant optical section thickness across all channels. The gain and offset of the photomultipliers was adjusted for each channel individually so that the observed fluorescence signal filled the linear range of the detectors, with the background being slightly positive and saturation being minimized.

### Image processing and quantitative colocalization studies

Image processing was performed using the Zeiss LSM Examiner software (version 3.2). Quantitative colocalization measurements were performed using the colocalization tool within the Zeiss LSM Examiner package. Four colocalization experiments were conducted: wtFc with EEA1 and with AC17, and hdFc with EEA1 and with AC17. For each experiment, we analyzed five optical sections from approximately five different regions, each containing 5–10 cells. Different optical sections within the same region were spaced 2 μm apart to ensure that each represented a unique image plane within the volume. Thresholds for the two channels in each image were set to be 2 standard deviations above the mean pixel intensity. Weighted colocalization coefficients (*M*) were calculated by summing the intensities of the above-threshold Fc-positive pixels that colocalized with above-threshold marker-positive pixels and dividing that number by the sum of all above-threshold Fc pixel intensities as follows:

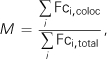

where Fc_i,coloc_ represents the intensity of a colocalized above-threshold pixel in the Fc channel, and Fc_i,total_ represents the intensity of any above-threshold pixel in the Fc channel. The value of *M* can range from 0 (no colocalization) to 1.0 (all pixels colocalize). The mean intensities of the labeled proteins were calculated to rule out that potential differences in colocalization coefficients resulted from higher levels of one labeled protein relative to another. The mean intensity values were wtFc and hdFc in the EEA1 colocalization: 1969 ± 342 and 2055 ± 713; wtFc and hdFc in the lamp-2 colocalization: 2088 ± 476 and 2346 ± 436; EEA1 in the wtFc and hdFc colocalizations: 1472 ± 414 and 1332 ± 454; lamp-2 in the wtFc and hdFc colocalizations: 1473 ± 629 and 1433 ± 396.

### Degradation assay

The apical surface of rFcRn-MDCK monolayers were incubated for 1 h with 1 μm [^125^I]labeled wtFc or hdFc at pH 5.9 as described for the immunofluorescence and transcytosis assays. Cells were cooled on ice and then washed four times with ice-cold pH 8.0 buffer. Prewarmed pH 8.0 buffer was added, and the cells were returned to 37°C for various times, after which cells were again cooled on ice. Media from the apical and basolateral surfaces were pooled and TCA precipitated, and the levels of radioactivity in TCA-soluble and TCA-insoluble fractions were determined. Cell-associated radioactivity was determined after lysis of cells in 0.1 N NaOH. The percent of an Fc ligand that was degraded at each time point was derived as [c.p.m._TCA-soluble_/(c.p.m._TCA-soluble_ + c.p.m._TCA-insoluble_ + c.p.m._cell-associated_)] × 100.

## References

[1] Rodewald R, Kraehenbuhl JP (1984). Receptor-mediated transport of IgG. J Cell Biol.

[2] Simister NE, Rees AR (1985). Isolation and characterization of an Fc receptor from neonatal rat small intestine. Eur J Immunol.

[3] Simister NE, Mostov KE (1989). An Fc receptor structurally related to MHC class I antigens. Nature.

[4] Simister NE, Story CM, Chen HL, Hunt JS (1996). An IgG-transporting Fc receptor expressed in the syncytiotrophoblast of human placenta. Eur J Immunol.

[5] Kristoffersen EK (1996). Human placental Fc gamma-binding proteins in the maternofetal transfer of IgG. APMIS Suppl.

[6] Leach JL, Sedmak DD, Osborne JM, Rahill B, Lairmore MD, Anderson CL (1996). Isolation from human placenta of the IgG transporter, FcRn, and localization to the syncytiotrophoblast: implications for maternal-fetal antibody transport. J Immunol.

[7] Ghetie V, Hubbard JG, Kim JK, Tsen MF, Lee Y, Ward ES (1996). Abnormally short serum half-lives of IgG in beta 2-microglobulin-deficient mice. Eur J Immunol.

[8] Israel EJ, Wilsker DF, Hayes KC, Schoenfeld D, Simister NE (1996). Increased clearance of IgG in mice that lack beta 2-microglobulin: possible protective role of FcRn. Immunology.

[9] Junghans RP, Anderson CL (1996). The protection receptor for IgG catabolism is the beta2-microglobulin-containing neonatal intestinal transport receptor. Proc Natl Acad Sci U S A.

[10] Raghavan M, Bjorkman PJ (1996). Fc receptors and their interactions with immunoglobulins. Annu Rev Cell Dev Biol.

[11] Burmeister WP, Huber AH, Bjorkman PJ (1994). Crystal structure of the complex of rat neonatal Fc receptor with Fc. Nature.

[12] Kim JK, Tsen MF, Ghetie V, Ward ES (1994). Localization of the site of the murine IgG1 molecule that is involved in binding to the murine intestinal Fc receptor. Eur J Immunol.

[13] Kim JK, Tsen MF, Ghetie V, Ward ES (1994). Catabolism of the murine IgG1 molecule: evidence that both CH2-CH3 domain interfaces are required for persistence of IgG1 in the circulation of mice. Scand J Immunol.

[14] Chaudhury C, Mehnaz S, Robinson JM, Hayton WL, Pearl DK, Roopenian DC, Anderson CL (2003). The major histocompatibility complex-related Fc receptor for IgG (FcRn) binds albumin and prolongs its lifespan. J Exp Med.

[15] Koltun M, Nikolovski J, Strong KJ, Nikolic-Paterson DJ, Comper WD (2004). Mechanism of hypoalbuminemia in rodents. Am J Physiol Heart Circ Physiol.

[16] Claypool SM, Dickinson BL, Yoshida M, Lencer WI, Blumberg RS (2002). Functional reconstitution of human FcRn in Madin-Darby canine kidney cells requires co-expressed human beta 2-microglobulin. J Biol Chem.

[17] Claypool SM, Dickinson BL, Wagner JS, Johansen FE, Venu N, Borawski JA, Lencer WI, Blumberg RS (2004). Bidirectional transepithelial IgG transport by a strongly polarized basolateral membrane Fcgamma-receptor. Mol Biol Cell.

[18] Yoshida M, Claypool SM, Wagner JS, Mizoguchi E, Mizoguchi A, Roopenian DC, Lencer WI, Blumberg RS (2004). Human neonatal Fc receptor mediates transport of IgG into luminal secretions for delivery of antigens to mucosal dendritic cells. Immunity.

[19] Dickinson BL, Badizadegan K, Wu Z, Ahouse JC, Zhu X, Simister NE, Blumberg RS, Lencer WI (1999). Bidirectional FcRn-dependent IgG transport in a polarized human intestinal epithelial cell line. J Clin Invest.

[20] McCarthy KM, Yoong Y, Simister NE (2000). Bidirectional transcytosis of IgG by the rat neonatal Fc receptor expressed in a rat kidney cell line: a system to study protein transport across epithelia. J Cell Sci.

[21] McCarthy KM, Lam M, Subramanian L, Shakya R, Wu Z, Newton EE, Simister NE (2001). Effects of mutations in potential phosphorylation sites on transcytosis of FcRn. J Cell Sci.

[22] Wu Z, Simister NE (2001). Tryptophan- and dileucine-based endocytosis signals in the neonatal Fc receptor. J Biol Chem.

[23] Kim KJ, Fandy TE, Lee VH, Ann DK, Borok Z, Crandall ED (2004). Net absorption of IgG via FcRn-mediated transcytosis across rat alveolar epithelial cell monolayers. Am J Physiol Lung Cell Mol Physiol.

[24] Martin WL, Bjorkman PJ (1999). Characterization of the 2:1 complex between the class I MHC-related Fc receptor and its Fc ligand in solution. Biochemistry.

[25] Ramalingam TS, Detmer SA, Martin WL, Bjorkman PJ (2002). IgG transcytosis and recycling by FcRn expressed in MDCK cells reveals ligand-induced redistribution. EMBO J.

[26] Detmer SA, Martin WL, Bjorkman PJ (2002). EMBO J.15:590–601. IgG transcytosis and recycling by FcRn expressed in MDCK cells reveals ligand-induced redistribution. EMBO J.

[27] Chaudhury C, Brooks CL, Carter DC, Robinson JM, Anderson CL (2006). Albumin binding to FcRn: distinct from the FcRn-IgG interaction. Biochemistry.

[28] Jones EA, Waldmann TA (1972). The mechanism of intestinal uptake and transcellular transport of IgG in the neonatal rat. J Clin Invest.

[29] Heldin CH (1995). Dimerization of cell surface receptors in signal transduction. Cell.

[30] Tuma PL, Hubbard AL (2003). Transcytosis: crossing cellular barriers. Physiol Rev.

[31] Huber AH, Kelley RF, Gastinel LN, Bjorkman PJ (1993). Crystallization and stoichiometry of binding of a complex between a rat intestinal Fc receptor and Fc. J Mol Biol.

[32] Sanchez LM, Penny DM, Bjorkman PJ (1999). Stoichiometry of the interaction between the major histocompatibility complex-related Fc receptor and its Fc ligand. Biochemistry.

[33] Schuck P, Radu CG, Ward ES (1999). Sedimentation equilibrium analysis of recombinant mouse FcRn with murine IgG1. Mol Immunol.

[34] Kim J, Bronson CL, Hayton WL, Radmacher MD, Roopenian DC, Robinson JM, Anderson CL (2005). Albumin turnover: FcRn-mediated recycling saves as much albumin from degradation as the liver produces. Am J Physiol Gastrointest Liver Physiol.

[35] Ober RJ, Martinez C, Vaccaro C, Zhou J, Ward ES (2004). Visualizing the site and dynamics of IgG salvage by the MHC class I-related receptor, FcRn. J Immunol.

[36] Janeway C (2005). Immunobiology. The Immune System in Health and Disease.

[37] Ober RJ, Martinez C, Lai X, Zhou J, Ward ES (2004). Exocytosis of IgG as mediated by the receptor, FcRn: an analysis at the single-molecule level. Proc Natl Acad Sci U S A.

[38] Monks J, Neville MC (2004). Albumin transcytosis across the epithelium of the lactating mouse mammary gland. J Physiol.

[39] Gastinel LN, Simister NE, Bjorkman PJ (1992). Expression and crystallization of a soluble and functional form of an Fc receptor related to class I histocompatibility molecules. Proc Natl Acad Sci U S A.

[40] Raghavan M, Chen MY, Gastinel LN, Bjorkman PJ (1994). Investigation of the interaction between the class I MHC-related Fc receptor and its immunoglobulin G ligand. Immunity.

[41] Nabi IR, Le Bivic A, Fambrough D, Rodriguez-Boulan E (1991). An endogenous MDCK lysosomal membrane glycoprotein is targeted basolaterally before delivery to lysosomes. J Cell Biol.

[42] Nabi IR, Rodriguez-Boulan E (1993). Increased LAMP-2 polylactosamine glycosylation is associated with its slower Golgi transit during establishment of a polarized MDCK epithelial monolayer. Mol Biol Cell.

[43] Fuller SD, Simons K (1986). Transferrin receptor polarity and recycling accuracy in “tight” and “leaky” strains of Madin-Darby canine kidney cells. J Cell Biol.

[44] Vaughn DE, Bjorkman PJ (1997). High-affinity binding of the neonatal Fc receptor to its IgG ligand requires receptor immobilization. Biochemistry.

[45] Martin WL, West AP, Gan L, Bjorkman PJ (2001). Crystal structure at 2.8 A of an FcRn/heterodimeric Fc complex: mechanism of pH-dependent binding. Mol Cell.

[46] Sanchez LM, Lopez-Otin C, Bjorkman PJ (1997). Biochemical characterization and crystalization of human Zn-alpha2-glycoprotein, a soluble class I major histocompatibility complex homolog. Proc Natl Acad Sci U S A.

[47] Apodaca G, Katz LA, Mostov KE (1994). Receptor-mediated transcytosis of IgA in MDCK cells is via apical recycling endosomes. J Cell Biol.

